# Stem transcriptome screen for selection in wild and cultivated pitahaya (*Selenicereus undatus)*: an epiphytic cactus with edible fruit

**DOI:** 10.7717/peerj.14581

**Published:** 2023-01-06

**Authors:** Omar Oltehua-López, Mario A. Arteaga-Vázquez, Victoria Sosa

**Affiliations:** 1 Universidad Autónoma Metropolitana Iztapalapa, Ciudad de México, Mexico; 2 INBIOTECA, Universidad Veracruzana, Xalapa, Veracruz, Mexico; 3 Biologia Evolutiva, Instituto de Ecologia AC, Xalapa, Veracruz, Mexico

**Keywords:** Transcriptomics, Domestication, Wild *vs* cultivars, Cacti, Dragon fruit, Drought tolerance, Pitaya, Maya, Epiphyte

## Abstract

Dragon fruit, pitahaya or pitaya are common names for the species in the *Hylocereus* group of *Selenicereus* that produce edible fruit. These Neotropical epiphytic cacti are considered promising underutilized crops and are currently cultivated around the world. The most important species, *S. undatus*, has been managed in the Maya domain for centuries and is the focus of this article. Transcriptome profiles from stems of wild and cultivated plants of this species were compared. We hypothesized that differences in transcriptomic signatures could be associated with genes related to drought stress. *De novo* transcriptome assembly and the analysis of differentially expressed genes (DEGs) allowed us to identify a total of 9,203 DEGs in the Hunucmá cultivar relative of wild Mozomboa plants. Of these, 4,883 represent up-regulated genes and 4,320, down-regulated genes. Additionally, 6,568 DEGs were identified from a comparison between the Umán cultivar and wild plants, revealing 3,286 up-regulated and 3,282 down-regulated genes. Approximately half of the DEGs are shared by the two cultivated plants. Differences between the two cultivars that were collected in the same region could be the result of differences in management. Metabolism was the most representative functional category in both cultivars. The up-regulated genes of both cultivars formed a network related to the hormone-mediated signaling pathway that includes cellular responses to auxin stimulus and to hormone stimulus. These cellular reactions have been documented in several cultivated plants in which drought-tolerant cultivars modify auxin transport and ethylene signaling, resulting in a better redistribution of assimilates.

## Introduction

Pitahayas are recognized as promising crops in areas with dry climates because they possess adaptations for occupying arid and semi-arid areas, and are creating fresh interest in view of global climate warming (Nerd, Tel-Zur & Mizrahi, 2002; Le Bellec, Vaillant & Imbert, 2006; Mizrahi, 2014; Sosa et al., 2020). The fruit of five species of Neotropical epiphytic cacti in the *Hylocereus* group of *Selenicereus* are known as dragon fruit, pitahaya or pitaya (*S. costaricencis, S. megalanthus, S. monacanthus, S. ocamponis* and *S. undatus*) (Nerd, Tel-Zur & Mizrahi, 2002; Le Bellec, Vaillant & Imbert, 2006; Mizrahi, 2014). Two species, *S. monacanthus* and *S. undatus*, were introduced to Southeast Asia and China and are currently widely cultivated around the world with Vietnam as the leading producer (Mizrahi, 2014; Rodríguez-Canto, 2015; Dahlin et al., 2017).

Pitahayas are considered valuable species, not only as edible fruits but also as a source of compounds such as betalains with beneficial antioxidant properties (Le Bellec, Vaillant & Imbert, 2006; Kamairudin et al., 2014). These components are derived from the pulp and pericarp, and also employed as colorants for food and cosmetics; moreover, the pulp contains abundant pectin (Nerd, Tel-Zur & Mizrahi, 2002; Jiménez-García et al., 2022; Pérez-Orozco & Sosa, 2022). *Selenicereus undatus* (Haw.) D.R. Hunt is the most widely cultivated species. It develops fruits with a reddish-purple pericarp and whitish pulp (Nerd, Tel-Zur & Mizrahi, 2002; Le Bellec, Vaillant & Imbert, 2006). This species is native to Mexico and Central America and some islands of the Lesser Antilles (Bauer, 2003; Barthlott et al., 2015; Sosa et al., 2020).

Historical evidence indicates that *S. undatus* was cultivated in pre-Columbian times on the Yucatan Peninsula, documented primarily by chroniclers who came to Mexico in the XVI century such as Diego de Landa who visited the Yucatan in 1560. Diego de Landa in his characterization of pitahayas described the reddish-purple color of the pericarp and the white pulp with small black seeds (Marcus, 1982; Rodríguez-Canto, 2015). Furthermore, it has been proposed that *S. undatus* was subjected to human selection before 3400 BC in the Maya domain (Colunga-García Marín & Zizumbo-Villarreal, 2004). Paleoethnobotanical analyses in the area of Chunchucmil from the Classic Maya period (A.D. 250–900) suggest that “solares” or homegardens comprised native fruit-bearing trees over which *S. undatus* was likely grown (Dahlin et al., 2017). Furthermore, pitahayas continue to be cultivated in homegardens or “solares” on the Yucatan Peninsula (Rico-Gray et al., 1990; De Clerck & Negreros-Castillo, 2000; Castro et al., 2018), as well as on small or large plantations (Cálix de Dios, Castillo-Martínez & Caamal-Canché, 2014).

Our focus is to identify genetic divergence among wild and cultivated plants of *S. undatus* by conducting a transcriptome screen of the stem. Our field work discovered several wild populations of this species in the lowlands of the Gulf of Mexico and on the Yucatan Peninsula. Transcriptomic characterization along with comparative genomics has brought about a revolution in the study of the processes involved in management and domestication in plants and animals (see review by Barrera-Redondo, Piñero & Eguiarte (2020)). Transcriptome research has been carried out in *Selenicereus megalanthus, S. monacanthus* and *S. costaricensis* to understand the effect of lighting at night on flowering, the effect of trypsin during storage of fruits (Pang et al., 2020), the response of roots to salt stress (Nong et al., 2019) and the response of plants to cold stress (Zhou et al., 2020) in addition to characterizing the metabolic pathways in betalain biosynthesis in fruits (Quingzhu et al., 2016; Cheng et al., 2020; Li et al., 2020; Hie et al., 2020; Zhang et al., 2021). Moreover, only a few genomes of species in *Cactaceae* have been assembled; in the case of *S. undatus* chromosome-scale or plastid genomes were based on cultivars from China such like “Yunnan” and “Guanhuabai” (Chen et al., 2021; Liu et al., 2021). These cultivars are the product of selection focusing on betalain biosynthesis and size of fruits (Chen et al., 2021; Liu et al., 2021). However transcriptomic research in cultivated *vs* wild *S. undatus* has not yet been carried out.

Mesoamerica, *i.e*. southeastern Mexico to the Northwest of Costa Rica, is an important center of plant domestication (Casas et al., 2007; Álvarez-Ríos et al., 2020). In this region, where *S. undatus* has been cultivated since pre-Columbian times, cultures have manipulated numerous plants using methodical selection, reproductive management techniques and other methods to select important attributes to be maintained in the population (Neto et al., 2016). Furthermore, a number of fruit species have been domesticated by the Maya (*e.g. Annona*: Larranaga et al., 2017; Caimito: Petersen, Parker & Potter, 2012; Gourd tree: Aguirre-Dugua et al., 2012; Huaya India: Jiménez-Rojas et al., 2019; Mexican plum: Fortuny-Fernández, Ferrer & Ruenes-Morales, 2017). Thus, in this study we explore whether there are unique transcriptomic signatures in the stems of wild and cultivated plants of *S. undatus* to gain insight into its management. Ecological-based niche modeling forecasted that *S. undatus* would have the largest predicted distribution, able to withstand dry and semi-dry climates (Sosa et al., 2020). Previous research discovered that variation in organs such as the roots and shoots of useful plants can be attributed to management for adaptation to intermittent drought (*e.g. Agave*: Huang et al., 2018; *Phaseolus*: Berny Mier y Teran et al., 2019; Rice: Han et al., 2022).

The objective of this article is to identify the transcriptomic signatures characteristic of wild and cultivated plants in stems of *S. undatus*, one of the most widely cultivated pitahaya species. Our hypothesis is that cultivated plants exhibit increased transcript levels of genes related to stress tolerance, in particular those involved in the response to hydric stress.

## Materials and Methods

### Plant materials

**Figure 1:**
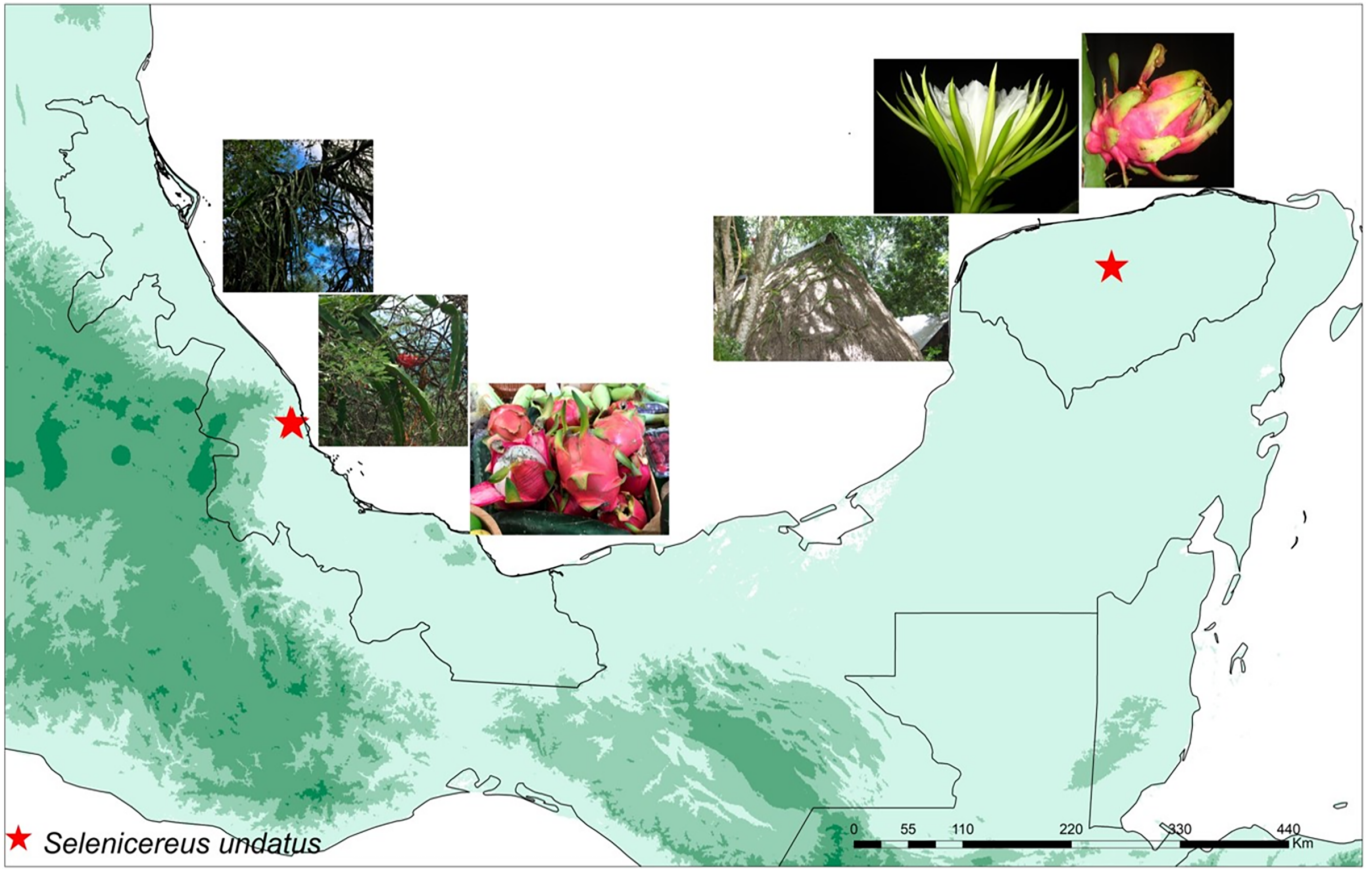
Localities of wild and cultivated plants. Map displaying the locations of the studied wild and cultivated plants of pitahaya (*Selenicereus undatus*). Wild plants were collected in Veracruz in the lowlands of the Gulf of Mexico in Mozomboa, Veracruz. Plants were growing over legume trees, the fruits are shown. The cultivated plants were collected in two homegardens located in Umán and Hunucmá in the Yucatan Peninsula. They were grown over trees in these agro-ecosystems; flower and fruits are displayed.

In total, eight samples were collected: four wild and four cultivated plants. Wild plants of *S. undatus* were collected in a ravine in Mozomboa, Veracruz in central Mexico, in the lowlands of the Gulf of Mexico. Plants were growing over legume trees in xerophytic shrubby vegetation, on a steep cliff (C. Ruiz, J. Ornelas & I Acosta 455 XAL) (MO1-, MO1-4, MO2A, MO2D) (Fig. 1). Cultivated plants were collected in two homegardens “solares” on the Yucatan Peninsula, in Hunucmá (HNC-2, HNC-3) and Umán (UMN1, UMN2), Yucatán (Ruiz-Domínguez, Ornelas & V. Sosa 498, 501, XAL) (Fig. 1). Plants were cultivated either on the stone walls that surround these agroecosystems or over legume trees. All collections were cultivated under the same greenhouse conditions for 8 weeks prior to RNA isolation, in a standard substrate (Fig. S1).

### Total RNA extraction

Stem tissue (200 mg) from four wild plants (Mozomboa) and two cultivated plants from the two different locations (Hunucmá and Umán) was used. The tissue was ground into a powder using a precooled mortar with liquid nitrogen. The ground powder mix was collected into a microcentrifuge tube and 1 ml TRizol Reagent (Thermo Fisher, Waltham, MA, USA) added. The tubes were vortexed for 30 s and centrifuged at 12,000*g* for 10 min at 4 °C. The supernatant was transferred into clean microcentrifuge tubes and added was an equal volume of phenol-chloroform-isoamyl alcohol (24:25:1 v/v). The solution was homogenized by vortexing for 30 s and centrifuged 13,000*g* for 15 min at 4 °C. The aqueous phase was collected in a clean tube and an equal volume of chloroform-isoamyl alcohol (24:25 v/v) added. The tubes were mixed thoroughly by inversion and centrifuged at 13,000*g* 10 min at 4 °C. The supernatant was recovered in a clean tube, and 750 μl of isopropyl alcohol was added to each tube. Then, the samples were incubated at room temperature for 10 min. After incubation, the tubes were centrifuged at 13,000*g* for 10 min at 4 °C to obtain pellets of RNA. Finally, the pellets were washed with 75% of EtOH in ultrapure RNase free deionized water treated with DEPC (diethyl pyrocarbonate) and centrifuged at 12,000*g* for 5 min at 4 °C. RNA was dissolved in RNase-free ddH2O and stored at −80 °C. Total RNA from five samples of each plant was used for library construction. RNA samples were processed by Labsergen (http://labsergen.langebio.cinvestav.mx/en/) for library preparation and sequencing. Libraries were sequenced using the Illumina NextSeq 500 system and paired-end sequencing.

### *De novo* transcriptome assembly and identification of differentially expressed genes (DEGs)

The Illumina NextSeq platform generated approximately 1,200 million reads. Before assembling the raw paired-end reads, we utilized Trimmomatic (ver. 0.40) (Bolger, Lohse & Usadel, 2014) for removal of adaptors and quality filtering (options: SLIDINGWINDOW:4:5 LEADING:5 TRAILING:5 MINLEN:25) (Bolger, Lohse & Usadel, 2014). The quality of raw reads was assessed by FastQC tools (http://www.bioinformatics.babraham.ac.uk/projects/fastqc). After cleaning and filtering the raw reads, they were assembled using Trinity (ver 2.13.1) (Haas et al., 2013) using default parameters for the paired-end assembly method to obtain the differentially expressed genes (Haas et al., 2013). Bowtie was used for reading the alignment against the *de novo* assembled reference transcriptome and RSEM (ver.1.3.3) (Li & Dewey, 2011) was used to estimate expression level values of alignments from each sample and among biological replicates (Li & Dewey, 2011). We used edgeR (R Bioconductor tools, Reilingen, Germany) (McCarthy, Chen & Smyth, 2012) for the identification of differentially expressed genes (DEGs). Normalization expression values were obtained by TMM normalization (Oshlack & Robinson, 2010). The sequencing data was deposited into NCBI database under ID BioProject PRJNA853281. We used the RnaSeqSampleSize method (Zhao et al., 2018), which is based on the distribution of average gene reads and dispersion from real RNA-seq data, to estimate power and determine if the sample size was sufficient.

### Functional annotation and classification

Functional annotation of the transcripts was obtained after the Trinity assembly was carried out with the Trinotate v.3.1.1 pipeline (http://trinotate.github.io). We used the set of open reading frames (ORFs) generated by TransDecoder v5.5.0 (TransDecoder. https://transdecoder.github.io/) to perform a protein-protein and transcript-protein search, BLASTP and BLASTX (e-value 1e−5) respectively, using public databases UniProt/Swiss-Prot protein (http://www.uniprot.org/). Protein domains were identified and annotated using HMMER v3.3 against the Pfam dataset (Finn, Clements & Eddy, 2011). Additionally, transmembrane regions were predicted using TMHMM v2.0 (Krogh et al., 2001); signal peptide prediction was determined using signalP v5.0 (Petersen et al., 2011). RNAmmer v1.2 was used to detect ribosomal RNA genes (Lagesen et al., 2007). Annotation outputs were reported using Trinotate SQLite database. The enrichment analysis was performed using the Cytoscape V3.8.0 (Shannon et al., 2003) and BiNGO (Biological Network Gene Ontology) (Maere, Heymans & Kuiper, 2005), with a hypergeometric statistical test and a *p*-value of 0.05. We also used the complete annotation file previously prepared with Trinotate as a custom reference. KEGG pathway analysis was performed by KASS (KEGG Automatic Annotation Server https://www.genome.jp/kaas-bin/kaas_main) using the BBH method (Moriya et al., 2007).

Transcriptome completeness was assessed using BUSCO v.3.0.1 (Benchmarking Universal Single-Copy Orthologs) to obtain the percentage of single-copy orthologues from two different data sets: viridiplantae_odb10 and eudicotyledons_odb10 (created in 2017).

We used the Plant Transcription Factor Database V5.0 (http://planttfdb.gao-lab.org/index.php) to assign DEGs to different transcription factor (TF) families by BLASTP and BLASTX (e-value 1e−5) against databases from three different plants belonging to the order Caryophyllales—the same order as pitahayas—(*i.e. Amaranthus hypochondriacus*, *Beta vulgaris* and *Dianthus caryophyllus)* and also from *Arabidopsis thaliana*, since it represents the best annotated angiosperm genome. The Transcriptome Shotgun Assembly project was deposited at DDBJ/EMBL/GenBank under the accession GKDI00000000. Annotations including gene ontology (GO) are included in Supplemental Material.

## Results

### Transcriptome sequencing, *de novo* assembly and functional annotation

A total 36,476,238 raw reads were obtained from eight libraries. After trimming the adaptors and filtering low-quality reads we obtained 29,662,120 (~81%) high-quality paired reads. A total of 406,766 transcripts and 230,996 assembled genes were identified with a median length of 240 base pairs (bp) and an N50 length of 941 bp. The assembled reference transcriptome was 204 Mb with a 43% of GC content. To evaluate the completeness of the assembly, we compared it against two different plant databases (viridiplantae and eudicotyledons) using BUSCO (Simão et al., 2015). Our results show that 56.1% of BUSCO genes are complete when compared against the viridiplantae set, 32.8% are fragmented and 11.1% are missing. From the comparison against the eudicot set, 55.2% are complete, 24.8% are fragmented and 20.0% are missing (Table S1). Based on the Trinotate pipeline, we were able to identify a total of 58,574 nucleotide sequences and 34,767 protein sequences that are related to different biological processes and molecular functions. In the full transcriptome assembly, the two most overrepresented terms within the category of molecular function are ATP binding and metal ion binding. While in the category of biological processes the four most overrepresented terms are photosynthesis, tricarboxylic acid cycle, glycolytic process and transcription DNA-templated (Fig. S2A). Two domains were found with higher frequency *in silico* translated transcripts: the protein kinase domain (PF00069.25/Pkinase) and the pentatricopeptide repeats (PF13041.6/PPR2), followed by leucine-rich repeat domain (PF13855.6/LRR_8, PF12799.7/LRR_4), RNA-binding domains (PF00076.22/RRM_1), WD domains (PF00400.32/WD40), as well as a p450 domain (PF00067.22/p450) (Fig. S2B).

### Analysis of DEGs

**Figure 2:**
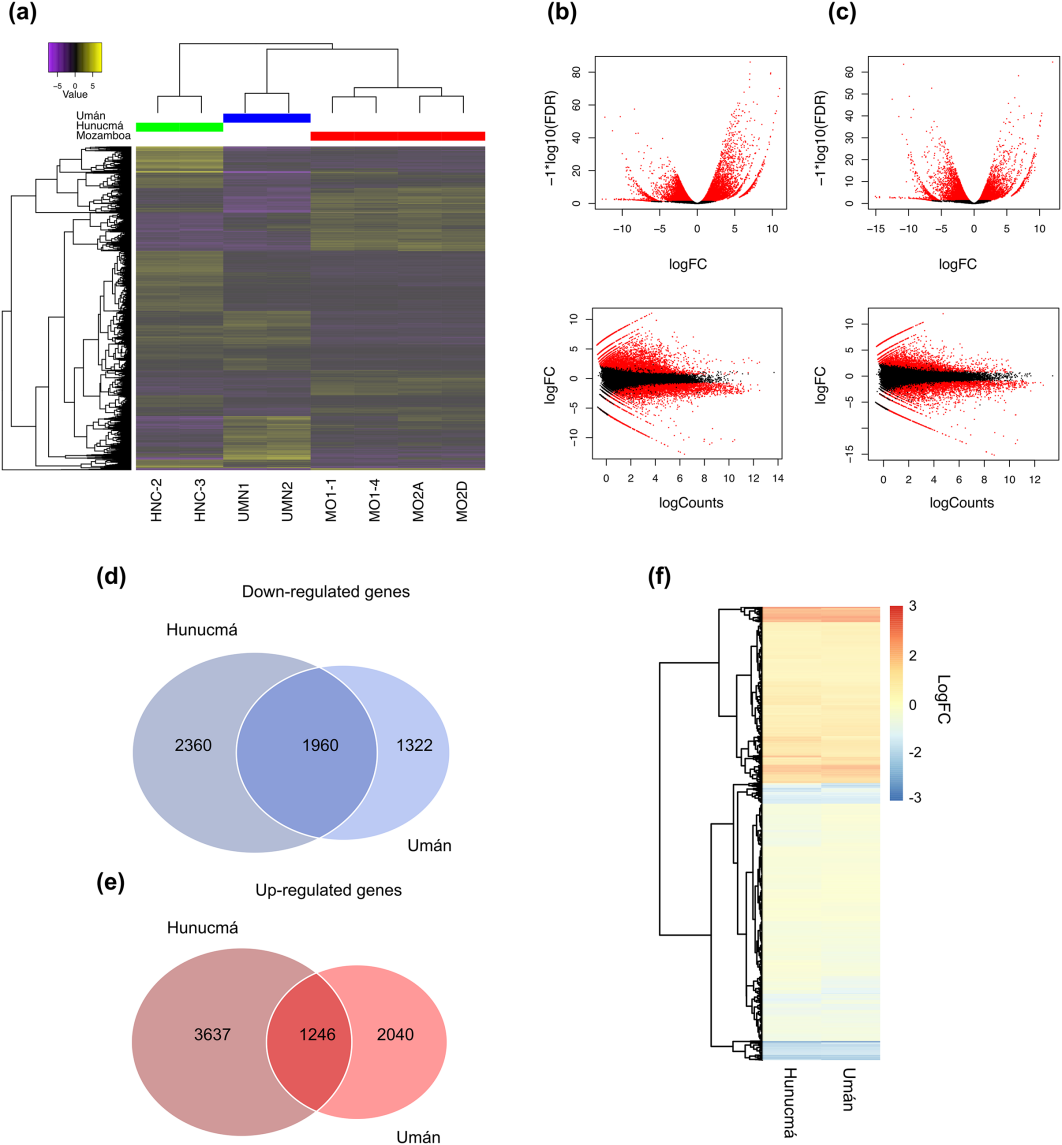
Overview of the differentially expressed transcripts from stems. Overview of the differentially expressed transcripts from stems of *Selenicereus undatus*. They were estimated in cultivated plants (Hunucmá and Umán) and in wild plants (Mozonboa). (A) Hierarchical clustering analysis of differentially expressed transcripts. Heatmap of relative expression levels for each transcript. The color key indicates the expression levels quantified as log2-FPKM transformation. The wild plants from Mozomboa (MO1-1, MO1-4, MO2A, MO2D), cultivated plants from Hunucmá (HNC-2, HNC-3) and Umán (UMN1, UMN2). (B) Venn diagram showing the number of up-regulated genes in Hunucmá and Umán cultivars. (C) Venn diagram showing the number of down-regulated genes in Hunucmá and Umán cultivars. (D) Heatmap of shared differentially expressed genes (1,246 up-regulated genes and 1,960 down-regulated genes) in cultivated plants from Hunucmá and Umán.

A total of 9,203 differentially expressed genes (DEGs) with a *p*-value (*p*) < 0.001 and a false discovery rate (FDR) < 0.01 were identified in the Hunucmá cultivar compared with wild plants of Mozomboa. DEGs were distributed as follows: 4,883 up-regulated and 4,320 down-regulated genes. We identified 6,568 DEGs in the Umán cultivar relative to wild plants, and of these, 3,286 correspond to up-regulated genes and 3,282 to down-regulated genes. Gene expression profiles from each sample are shown in a heatmap plot (Fig. 2A). These results show differences between the two cultivated plants. The heatmap and the Pearson’s correlation showed clustering between the replicas for each of the samples and the groups of samples distributed between the two PCs; PC1 represents 36.15% of the variance and PC2 represents an additional 17.78%. When we compared the two sets of DEGs to detect the genes shared among the cultivated plants, we identified 1,246 up-regulated and 1,960 down-regulated genes (Figs. 2B, 2C). The expression profile of DEGs among cultivated plants is very similar (Fig. 2D). Similarity among wild and cultivated plants in a matrix heatmap is shown in Fig. S2. Distribution of DEGs in the cultivars is shown in Fig. S3. The statistical power of this experimental design calculated in RnaSeqSampleSize is 0.57.

### DEGs: functional classification

**Figure 3:**
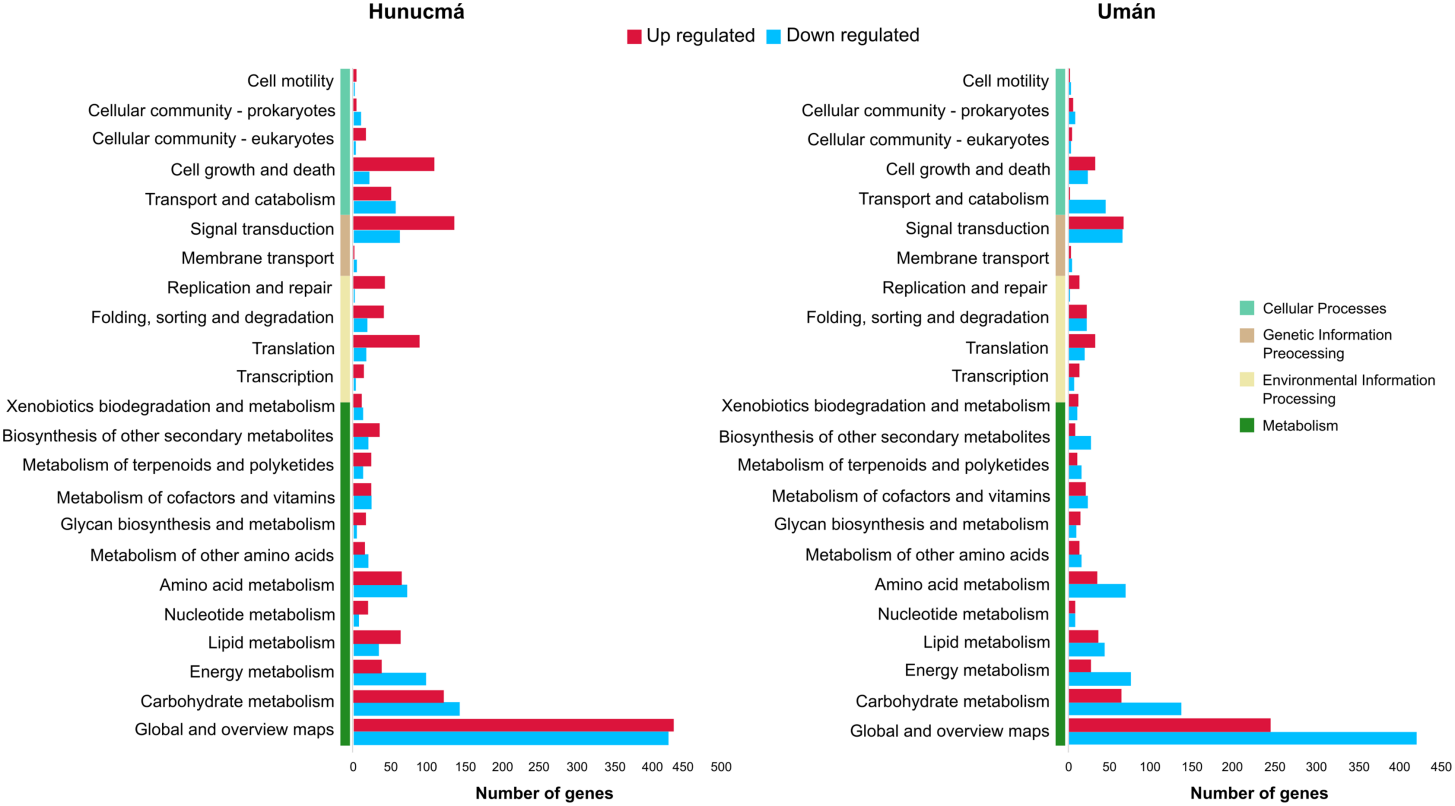
Functional classification of differentially expressed genes (DEGs) in the Hunucmá and Umán cultivated plants. Barplots indicate the KEGG metabolic pathway annotation of DEGs across four different categories.

A total of 2,469 genes from Hunucmá and 1,716 genes from Umán were assigned to the categories: *metabolism*, *environmental information processing*, *genetic information processing and cellular processes*. The profiles of down-regulated genes are very similar in both cultivated plants; Hunucmá has 1,076 genes and Umán 1,042 genes distributed in different categories. However, the profiles of up-regulated genes differ for the cultivated plants: Hunucmá has 1,393 genes, while Umán only has 674 genes distributed across all categories. The main differences can be observed in: *cell growth and death* and *signal transduction*. The most overrepresented category in both cultivated plants is *metabolism* with 1,750 genes from Hunucmá and 1,334 genes from Umán, and within this category 858 and 665 genes from Hunucmá and Umán respectively, are located in *global and overview maps*, where the most representative modules are *metabolic pathways* and *biosynthesis of secondary metabolites*. For the *carbohydrate metabolism* pathway, there are 265 and 200 Hunucmá and Umán genes, respectively (Fig. 3).

GO functional annotation analysis of DEGs showed that in the *biological process* category the *response to stress* and *response to stimulus* are the most significant term for down-regulated genes in both cultivated plants, whereas in the up-regulated genes the enriched GO terms in Hunucmá and Umán are related with *cellular process* and *signaling*. This last term is connected to *signaling pathway* and *hormone-mediated signaling pathway*, which in turn interact with *cellular response to hormone stimulus* and *auxin mediated signaling pathway*. In the *molecular function* category, the down-regulated genes are related to *catalytic activity*, *enzyme regulator activity* and *hydrolase activity* in both cultivated plants. Nevertheless, the up-regulated genes are involved in *binding*, a term which is composed of a subset of terms like *binding nucleoside* and *binding nucleotide*. The *hydrolase activity* pathway is also present in up-regulated genes connected by *catalytic activity* term (Figs. S4 and S5).

### DEGs: annotation of transcription factors families

**Figure 4:**
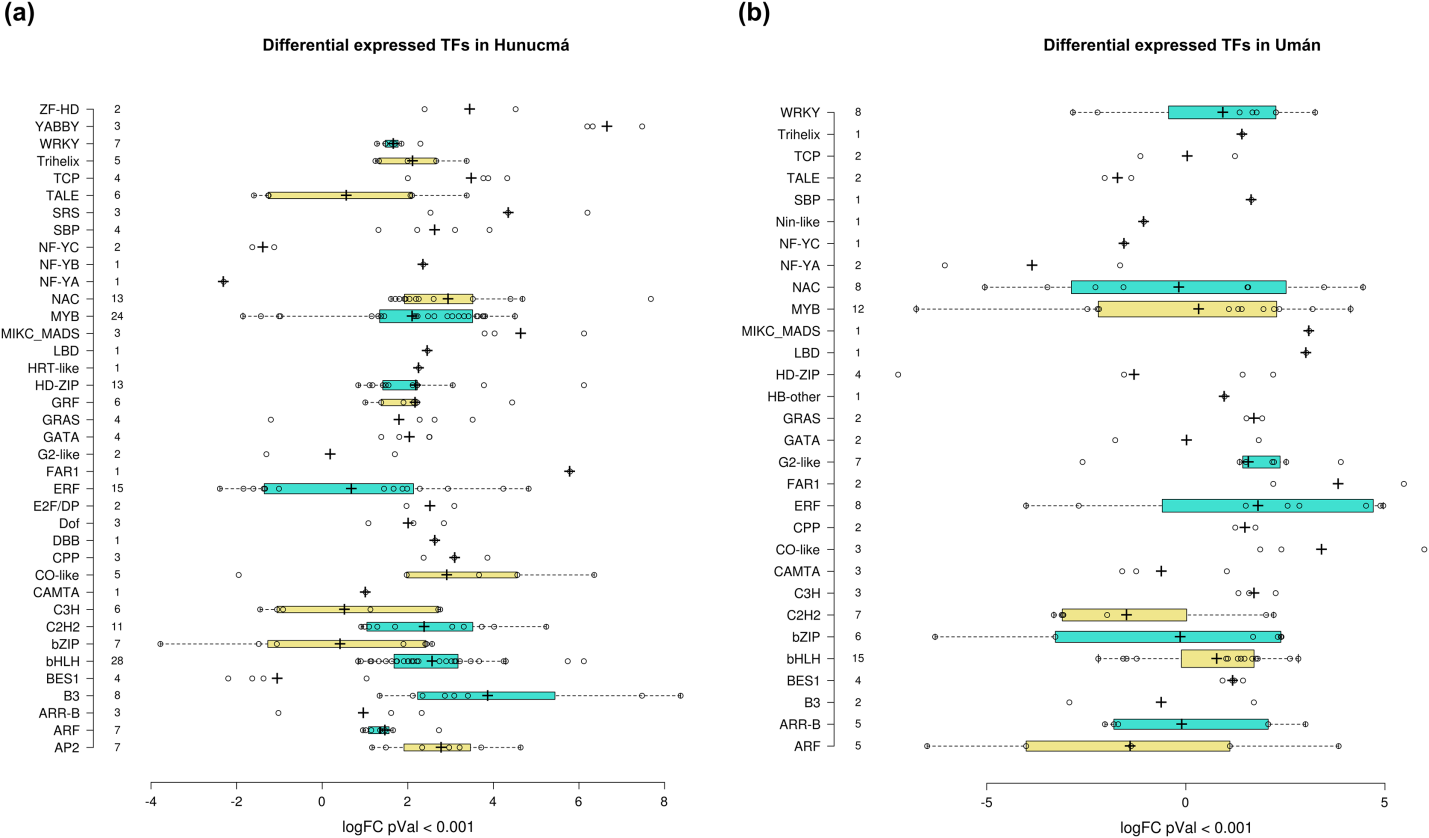
Transcription factor families differentially expressed in cultivated plants. Boxplots show of log2FC value distribution of differentially expressed TFs with *p*Val < 0.001. The box are proportional to square-roots of the numbers of TFs per family; the crosses indicate the mean of the samples; the box limits indicate the 25th and 75th percentiles as determined by R software (http://shiny.chemgrid.org/boxplotr/). Tukey test to define the whiskers extended in 1.5 times the interquartile range from the 25th and 75th percentiles; the open circles represent the data points and the number of sample points are on the y-axis.

Transcription factors (TFs) are proteins that play an important role during the regulation of gene expression. A total of 221 and 121 DEGs of Hunucmá and Umán, respectively, were identified as TFs (Figs. S6, S7): 38 families were identified in Hunucmá and 30 families in Umán. Most of the DEGs identified as TFs are members of the *bHLH* (basic helix-loop-helix), *MYB* (myeloblastosis), *ERF* (ethylene response factor), *NAC* [*NAM* (no apical meristem, Petunia), ATAF1–2 (*Arabidopsis thaliana* activating factor), and *CUC2* (cup-shaped cotyledon, Arabidopsis)], *C2H2* (Cys2-His2, zinc finger proteins), *HD-ZIP* (homeodomain-leucine zipper), *WRKY* (*WRKYGQK* domain), *bZIP* (basic leucine zipper) and *ARF* (auxin response factor), *CAMTA* (calmodulin-binding transcription activator) families, among others. The TF family with the highest number of DEGs in Hunucmá is the *bHLH* family with 28 up-regulated genes and in Umán with 11 up-regulated and four down-regulated genes. The *bHLH* proteins play an important role in the regulation of growth and development, as well as the response to stress in plants. The second family of TFs with the highest number of DEGs is the *MYB* family with 20 up-regulated and four down-regulated genes in Hunucmá, while in Umán there were eight up-regulated and four down-regulated *MYB* genes. Hunucmá has 100 more DEGs than Umán, identified as TFs and due to this result other TF families with more than 10 DEGs can be observed in Hunucmá. *ERF* family has the third most DEGs with 13, followed by *NAC* and *HD-ZIP* with 13 and *C2H2* with 11 DEGs (Fig. 4).

**Figure 5:**
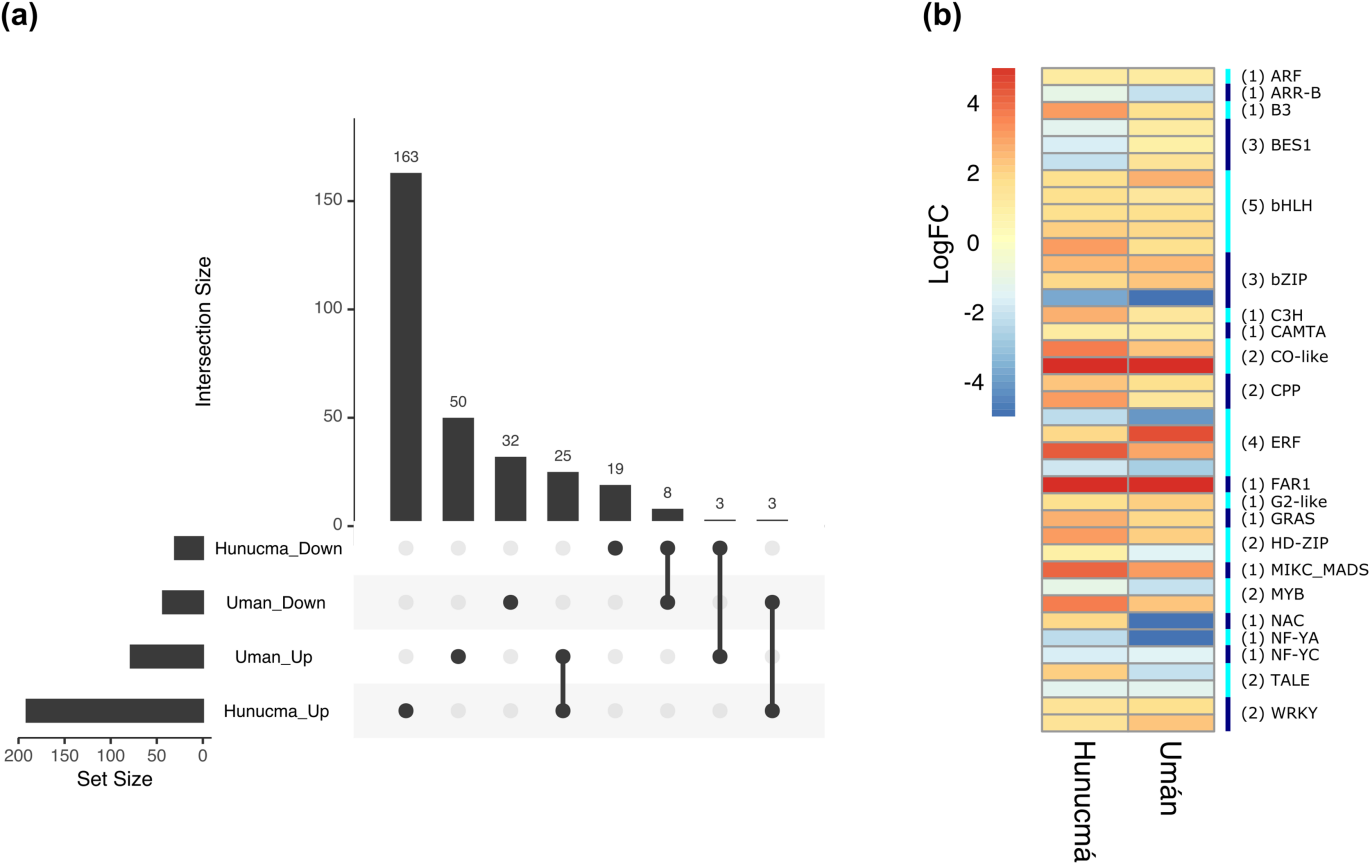
Differentially expressed transcription factors (TFs) shared between Hunucmá and Umán plants. (A) UpSet diagram showing shared and unique differentially expressed TFs between Hunucmá and Umán plants, the diagram shows the unique TFs in each cultivated plants. (B) Expression levels of the TFs that are shared in both cultivated plants. The right side bar shows the rows corresponding to the genes per TFs family identified in both cultivated plants.

The DEGs shared between the two cultivated plants identified a total of 39 associated with 22 different families of TFs. Twenty-five genes are up-regulated and eight down-regulated, while six have different expression profiles (Fig. 5A). The two families with the highest number of DEGs are *bHLH* with five and *ERF* with four. All the genes identified as *bHLH* are up-regulated, while among the four genes in the *ERF* family, two are up-regulated and two are down-regulated (Fig. 5B). Different members of the *ERF* family have been reported as key regulators of various stress responses in plants (Mizoi, Shinozaki & Yamaguchi-Shinozaki, 2012; Xie et al., 2019). Other TF families involved in the response to different types of stress identified with DEGs shared between the two cultivated pitaya plans are *WRKY*, *NAC*, *MYB* and *CAMTA*.

## Discussion

### DEGs: differentially expressed genes

Transcriptomic analyses of this project found differences in gene expression levels from the stems of two cultivated plants compared to wild-type plants of *S. undatus*. This data introduces basic transcriptional information for further research on cacti that produce edible fruit. Interestingly, 2.11% of the genes increased their expression in the stems of cultivated plants from Hunucmá and 1.42% of the Umán cultivar from homegardens, compared to the stems of wild plants. In contrast, 1.87% of the genes decreased their expression in Hunucmá and 1.42% in Umán plants. A number of studies of edible fruit have shown that using either transcriptomic screening or other molecular methods it is possible to understand divergence in genes from cultivated and wild plants, as has this study (*e.g*. Chichipe: Otero-Arnaiz et al., 2005; *Stenocereus*: Parra et al., 2010; Ruán-Tejeda et al., 2014; Loquat: Jiang et al., 2016; Strawberry: Qiao et al., 2016, Breadfruit: Laricchia et al., 2018; Prickle pear: López-Palacios et al., 2018; Chia: Peláez et al., 2019; Eggplant: Page et al., 2019; Persimmon: Guan et al., 2017).

### DEGs: functional classification and family genes

Approximately half of the DEGs are shared between the two cultivated plants and probably the rest of the differences between the cultivars might be related to human management over time. Our results indicate that *metabolism* was the most representative category according to the functional classification of KEGG (the database for understanding high-level functions and utilities of the biological system: https://www.genome.jp/kegg/) and similar patterns of down-regulated genes were found between wild and cultivated plants. In contrast, expression levels of genes related to *cellular processes* are higher in Hunucmá than in Umán plants.

Interestingly, one group of shared genes shows a very similar pattern of expression. We identified that the shared up-regulated genes in the category of *biological process* are enriched in terms of signaling pathway, which in turn are connected with the *hormone-mediated signaling pathway* that includes *cellular responses to auxin stimulus* and *cellular responses to hormone stimulus*. These cellular reactions have been documented in several cultivated plants such as barley, in which drought-tolerant cultivars modify auxin transport and ethylene signaling, resulting in a better redistribution of assimilates (Hong, Ni & Zhang, 2020; Qiu et al., 2020). Furthermore, evidence of selection of drought-resistance genes has been documented in cultivated plants such as weedy rice (Han et al., 2022), cotton (Shim, Bandillo & Angeles-Shim, 2021) and moth bean (Yundaeng et al., 2019). Thus, the enrichment of genes found in the cultivated plants of pitahaya might be related to the response to drought.

Worldwide, abiotic stress is one of the main limitations for growth and development of crops (Dresselhaus & Hückelhoven, 2018). Moreover, plants can respond to abiotic stress using different strategies that include transcriptional control of different gene networks through the expression of transcription factors (He et al., 2016; Arisha et al., 2020). Dragon fruits are epiphytic cacti which can grow under different environmental conditions, including areas under dry conditions, due to their high efficiency in water consumption and reduction of water loss due to their CAM photosynthesis, which allows them to assimilate carbon with the stomata closed during the day (Nerd, Tel-Zur & Mizrahi, 2002). In plants with CAM photosynthesis, some families of transcription factors that change their expression under drought stress have been recorded. For example, *Agave sisalana* subjected to drought stress, shows an increase in the expression of the transcription factor families *MYB*, *AP2/ERF* and *bHLH* (Sarwar et al., 2018). Also, in *Cynanchum thesioides* a plant from the arid and semi-arid areas of Asia, hundreds of DEGs were identified under severe drought stress including transcription factors belonging to the *MYC* and *ERF* families (Zhang et al., 2019).

### DEGs: transcription factor families

TFs such as *bHLH, NAC, B21P, BH21P* related to abiotic stress were overexpressed in the cultivated plants from the homegardens of the Yucatan in comparison with the wild type plants from Veracruz. Furthermore, there were differences in expression of these TFs between the cultivars from Umán and Hunucmá, which are located in the same region of the Yucatan. TF divergences in domesticated plants have been encountered in many species, in particular to plants grown under abiotic stress such as chickpea (Moenga et al., 2020) or the ramie (Li et al., 2014). Therefore, it can be hypothesized that the origin and management of the plants in Hunucmá and Umán has been different. TFs related to biotic stress are adaptive, and moreover from our observations of the cultivated collections under greenhouse conditions prior to extracting RNA, more secondary roots and secondary growth in Hunucmá plants were observed in comparison with the Umán plants. Additional samples of cultivated pitahayas on the Yucatan Peninsula and additional transcriptomic research can bring a deeper understanding of the management of *S. undatus* by the Maya.

## Importance and conclusions

*De novo* transcriptome assembly has evolved as a resourceful and effective approach for answering evolutionary questions (Hölzer, 2020). Functional genomics resources broaden the knowledge of the genes linked to horticultural and agronomically important traits (Rai & Shekhawat, 2014). Transcriptomic research has been conducted on *S. megalanthus, S. costaricensis* and *S. monacanthus* to address a number of physiological processes. For instance, transcriptomic research has investigated the effect of lighting at night, the trypsin effect in fruit storage (Pang et al., 2020), the response of roots to salt stress (Nong et al., 2019), the response of plants to cold stress (Zhou et al., 2020), and to characterize metabolic pathways in betalain biosynthesis in fruits (Quingzhu et al., 2016; Cheng et al., 2020; Li et al., 2020; Hie et al. 2020). Recently, for *S. undatus*, a chromosome level genome found genome duplication of betacyanin biosynthetic genes (Zheng et al., 2021).

The totality of the transcriptomic and genomic research in *S. costaricensis, S. monacanthus*, and *S. undatus* has been conducted in plants cultivated in Asia, mostly in China, where large plantations provide produce for regional markets (Yu et al., 2021). The aim of the research conducted here was to compare the transcriptomic signal in wild vs cultivated plants of *S. undatus* in their native range with confidence in the origin and identification of plants.

Our analyses were performed before the release of the genome of *S. undatus* (Zheng et al., 2021), therefore we used *de novo* transcript assembly. This research is the first to investigate transcriptomic signal in stems of wild plants of *S. undatus*. Plants were collected in the north of the Gulf of Mexico Province, in ravines with tropical dry forests, growing over several legume trees in areas with annual average temperature of 30 °C and annual precipitation of 1,500 mm (Sosa et al., 2020). In contrast, cultivated plants were collected in the Maya domain, in the Yucatan Peninsula, in “solares” or homegardens where they have been managed and cultivated for centuries (Colunga-García Marín & Zizumbo-Villarreal, 2004). Pitahayas are commonly grown by the Maya in the Peninsula over stone walls without shade of trees where annual temperature is higher, up to 35 °C (Castro et al., 2018). *S. undatus* is planted as well in semiarid regions in the north of Mexico in zones with annual precipitation of 690 mm (Osuna-Enciso et al., 2016). Wild and cultivated plants utilized here were maintained under the same greenhouse conditions for 3 months before conducting our research to identify transcriptomic signatures. Therefore, we expected to find differences in genes related to the plants’ ability to withstand drier habitats comparing wild and cultivated plants. Our results indicate that there are differences in TFs related to biotic stress, in wild and even between cultivated plants. Root and fruit transcriptomics will be further investigated to gain a broader perspective on how people in Mexico have managed this pitahaya, and to be able to propose new strategies and germplasm for cultivating this economically important species.

Fruit crops can provide health and nutrition, they are a rich source of carbohydrates, fats, proteins, energy, vitamins and minerals, as well as dietary fiber. They can prevent various diseases, including diabetes, anemia, and hypertension. They have the potential to provide a source of income and can be sold fresh or in value-added products (Meldrum et al., 2018). There are many neglected and underutilized species that have potential to diversify not only the human diet, but also to increase food production levels, and thus make more sustainable and resilient agro-systems possible (Baldermann et al., 2016). Therefore, research on plants such as pitahayas reveals alternatives to healthy and functional diets with increased value since not only the fruit, but also the by-products of the peels have potential phytochemical benefits.

## Supplemental Information

10.7717/peerj.14581/supp-1 Supplemental Information 1 Supplementary figures and tables.

10.7717/peerj.14581/supp-2 Supplemental Information 2 Annotated Differentially Expressed Genes (DEGs).

10.7717/peerj.14581/supp-3 Supplemental Information 3 Functional annotation of the transcripts by Trinity assembly carried out with Trinotate pipeline.

## Additional Information and Declarations

### DNA Deposition

The following information was supplied regarding the deposition of DNA sequences: The sequencing data is available at NCBI PRJNA853281. The Transcriptome Shotgun Assembly project is available at DDBJ/EMBL/GenBank: GKDI00000000.

### Data Availability

The following information was supplied regarding data availability: The molecular data is available at figshare: Oltehua-López, Omar; Sosa, Victoria; A. Arteaga-Vazquez, Mario (2022): data_VS_SUn. figshare. Dataset. https://doi.org/10.6084/m9.figshare.20173444.v1
