# Supplementary material for: Stem transcriptome screen for selection in wild and cultivated pitahaya (Selenicereus undatus): an epiphytic cactus with edible fruit
Source: PeerJ. 2023 Jan 6;11:e14581. doi: 10.7717/peerj.14581 (PMC9828283; doi:10.7717/peerj.14581)
Supplement: Supplemental Information 1 [file peerj-11-14581-s001.pdf]

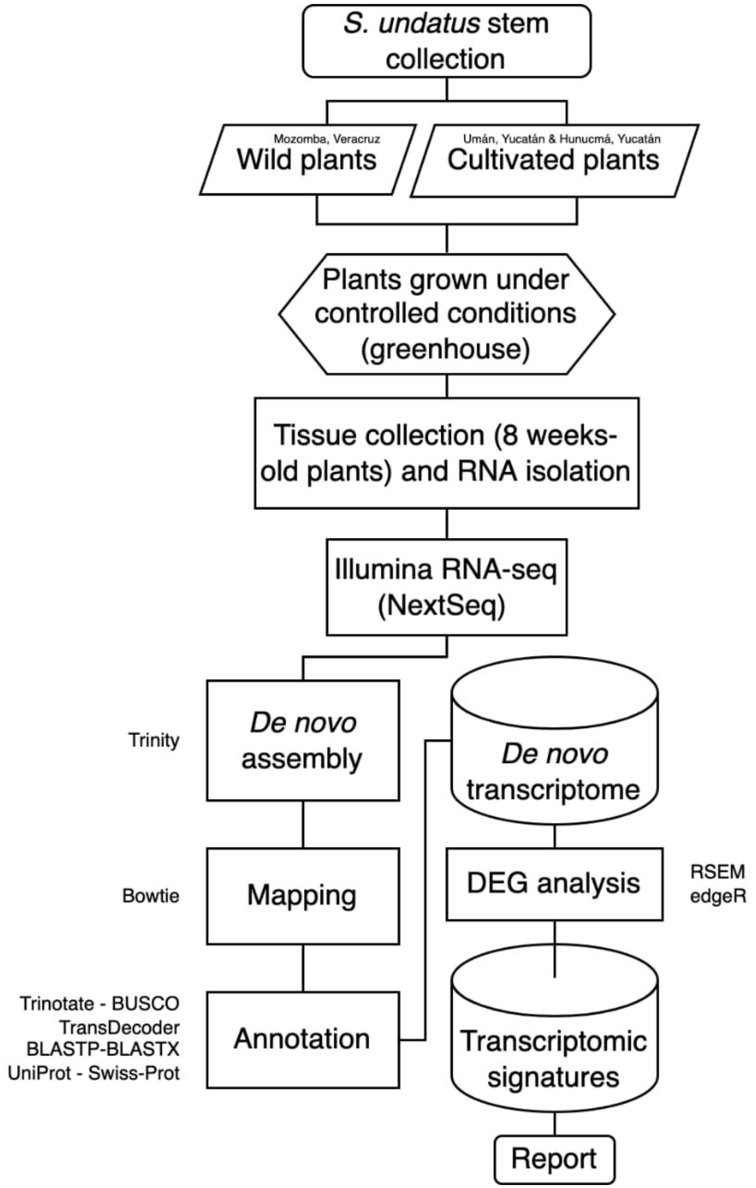

Figure S1: Work flow for sampling, greenhouse conditions and data analysis steps of the *de novo* transcriptome in *Selenicereus undatus*.

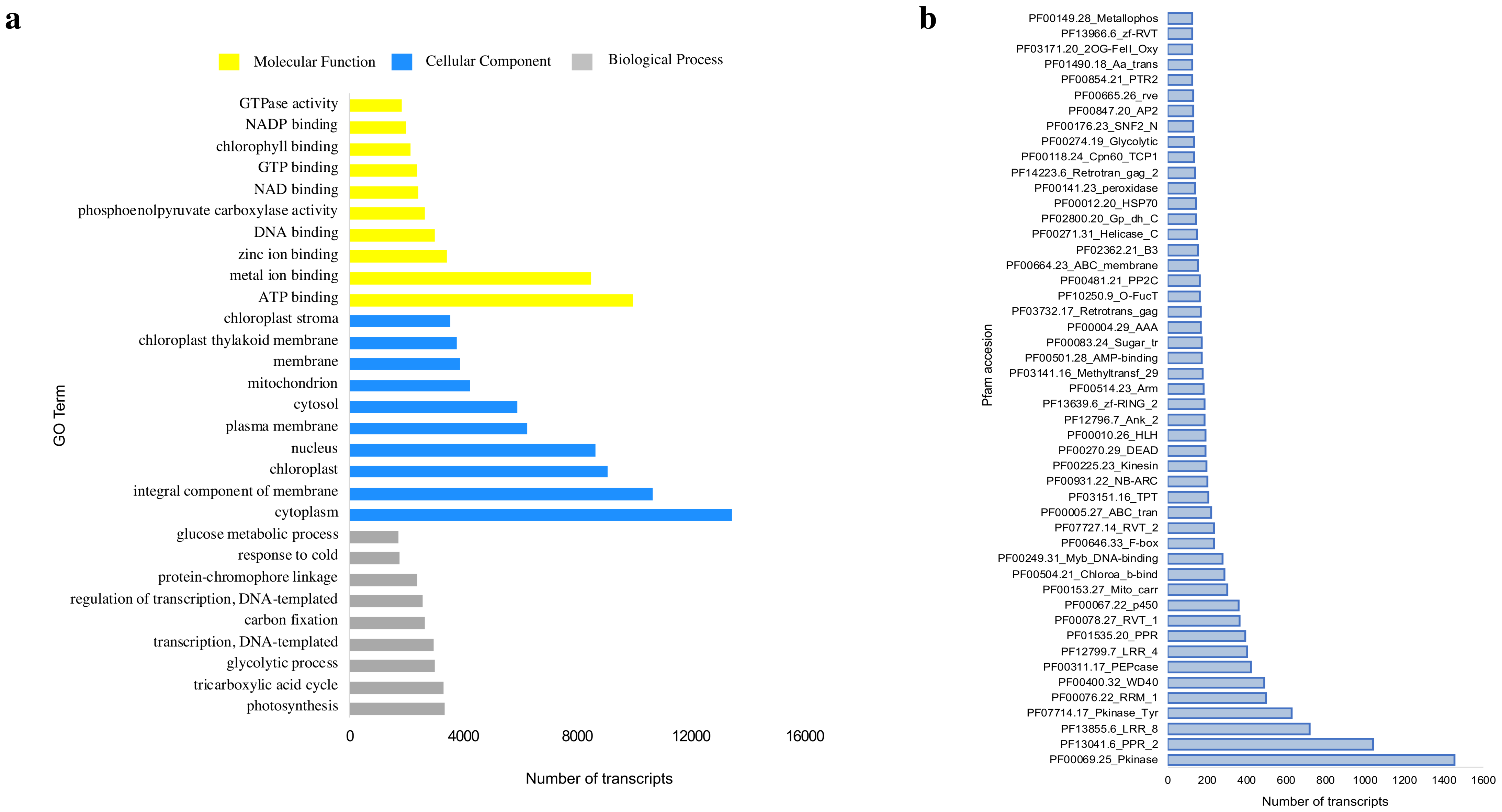

Figure S2: Distribution of *de novo* transcripts. a) Top ten gene ontology (GO) terms from transcriptome annotation, results for Molecular Function, Cellular Component and Biological Process. b) Top fifty distribution of Pfam domains found in the transcriptome (independent E-value < 10–5 were considered).

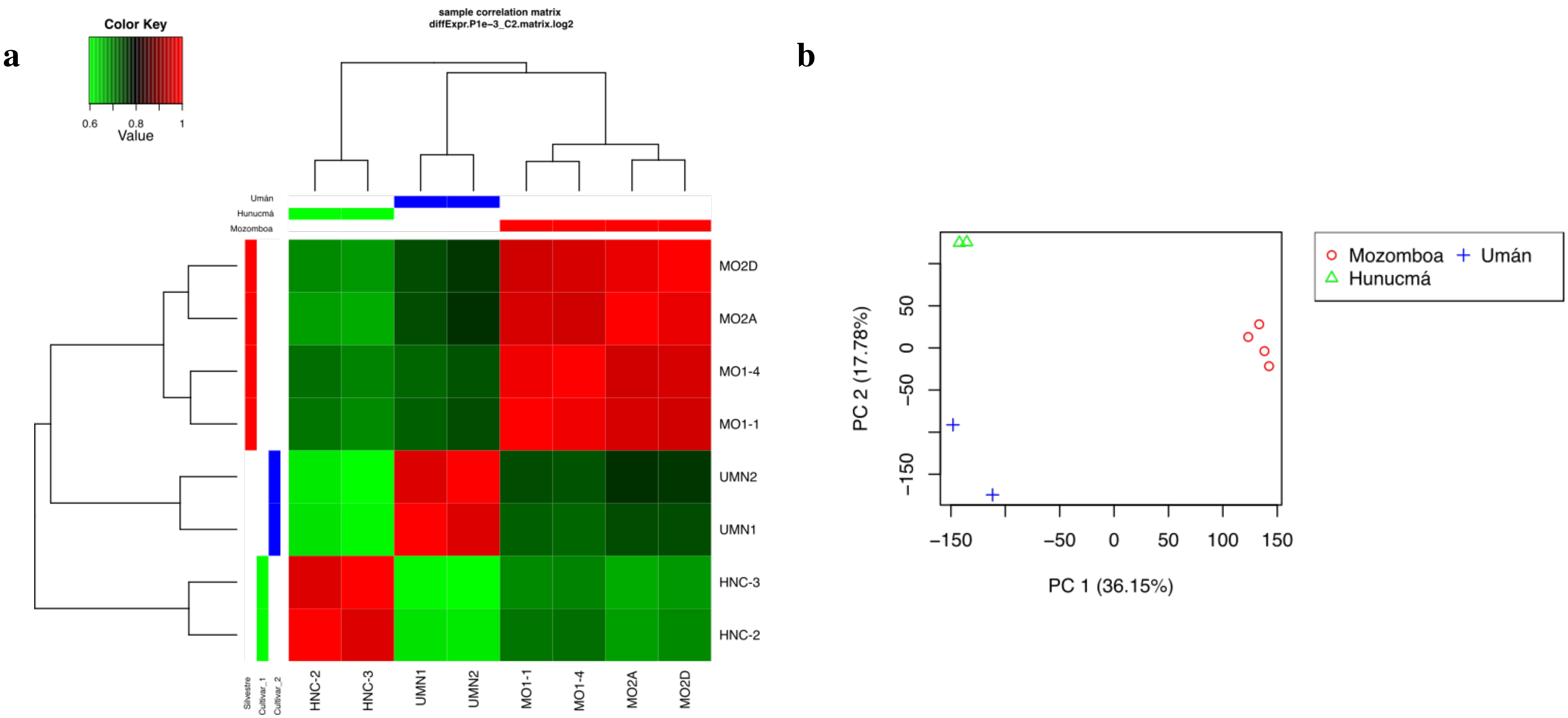

Figure S3: Comparative analysis of similarity across replicates of each sample.
a) Correlation matrix heatmap show the Pearson's correlation values (pairwise comparison) betweeen Mozomboa wildtype plants (red bar) and the two cultivars Hunucmá (green bar) and Umán (blue bar). b) Principal Component Analysis (PCA) displaying the similarity among samples.

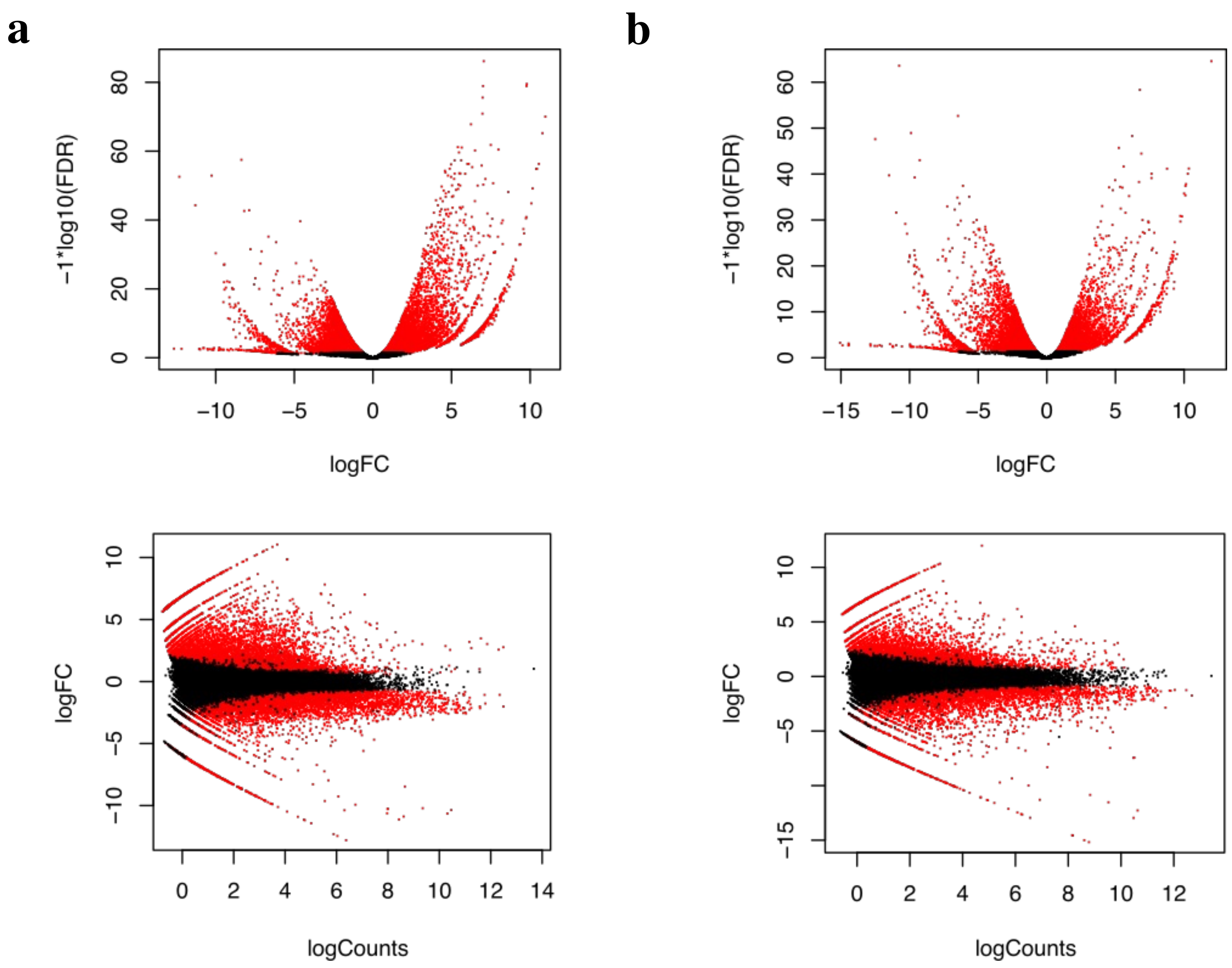

Figure S4: Distribution of differentially expressed genes identified (DEGs) in cultivars and wild type plants. a) Volcano plot (top row) and MA plots (bottom row) of DEGs between Hununcmá and Mozamboa plants. b) Volcano plot (top row) and MA plots (bottom row) of DEGs between Umán and Mozamboa plants.

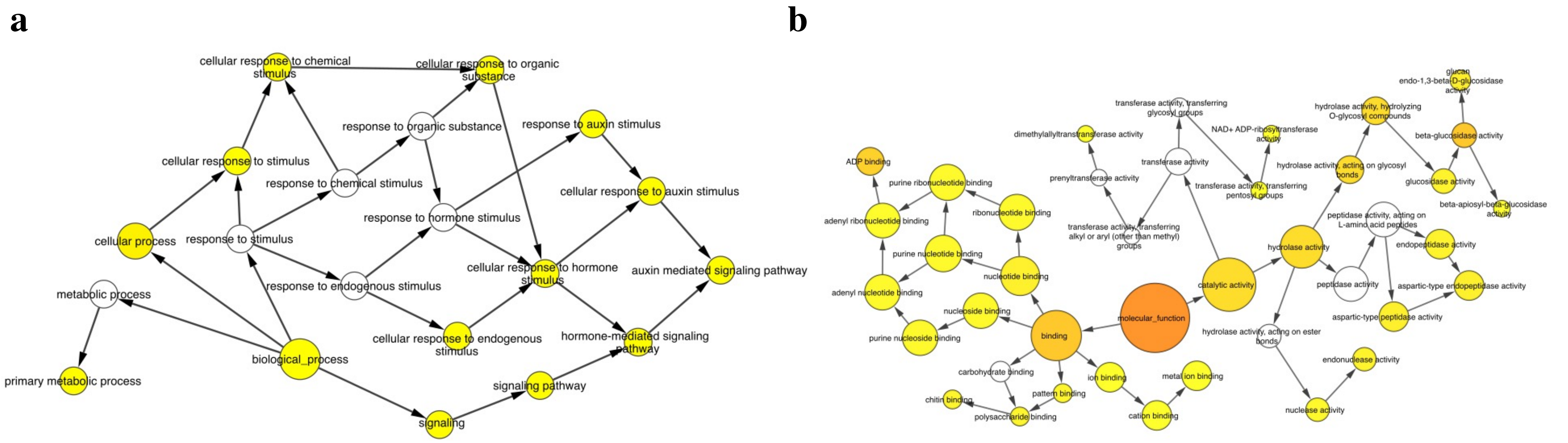

Figure S5: GO overrepresentation analysis with Up-regulated shared genes. a) Enrichment of GO terms in biological process by BiNGO (adjusted p-value < 0.05) and molecular function b) The size and color of each node indicate the number of enriched GO terms.

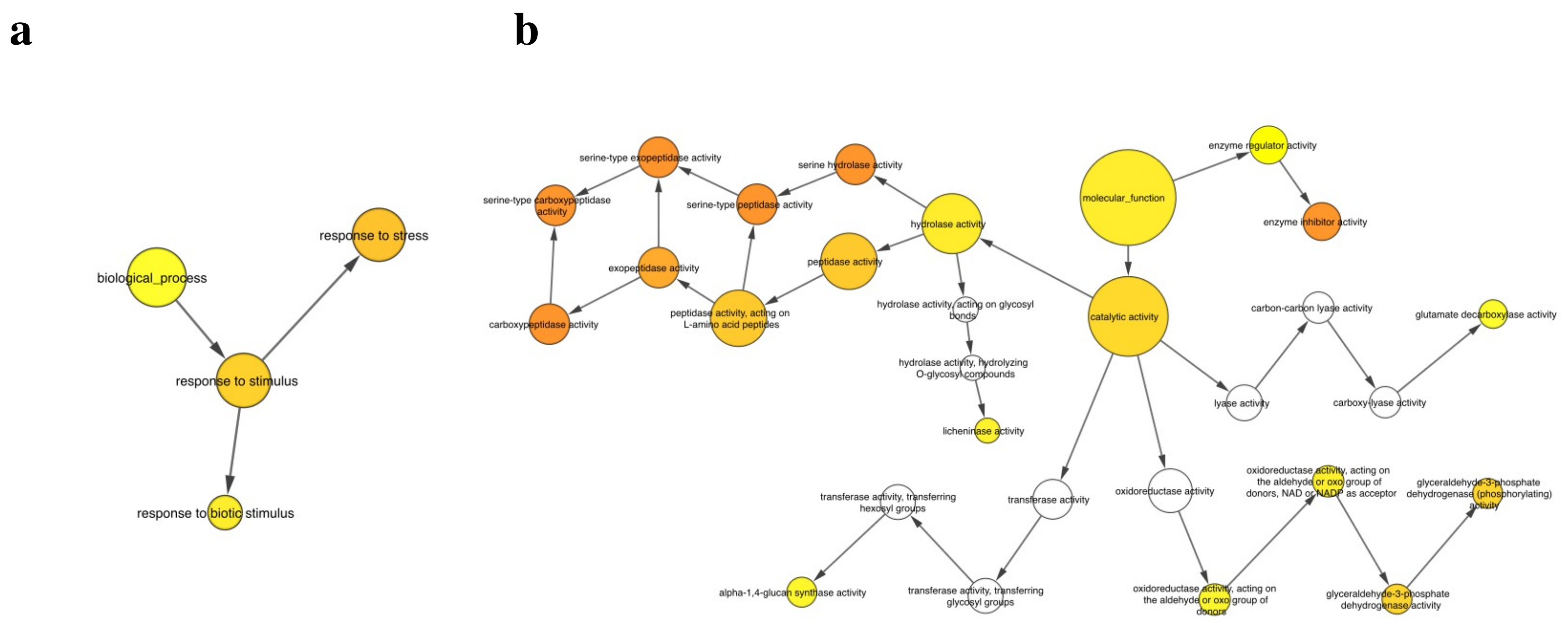

Figure S6: GO overrepresentation analysis with Down-regulated shared genes. a) Enrichment of GO terms in biological process by BiNGO (adjusted p-value < 0.05) and molecular function b) The size and color of each node indicate the number of enriched GO terms.

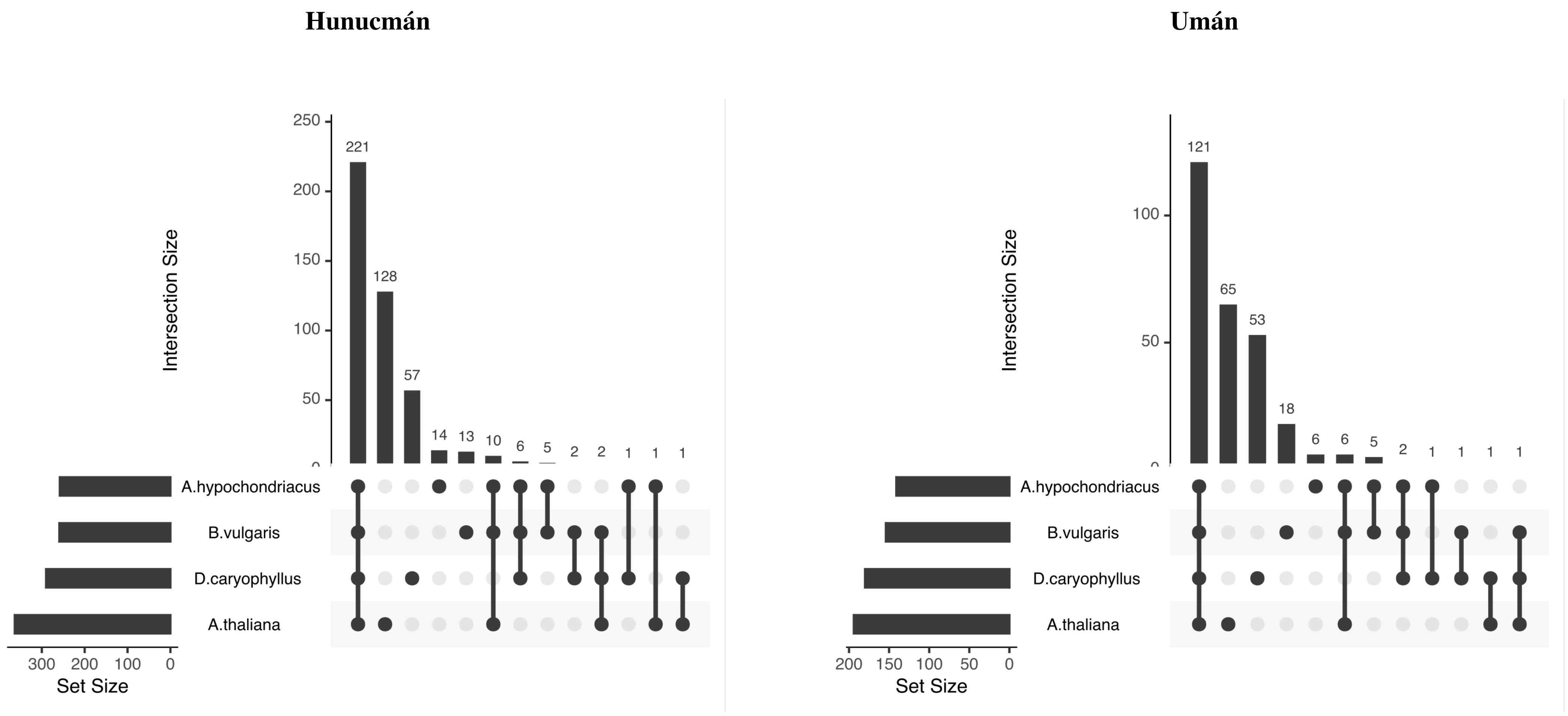

Figure S7: Distribution of BLASTX and BLASTP results in best hit among different species.

Table S1 Summary of BUSCO statistics from the *Selenicereus undatus* assembled reference transcriptome compared to eudicotyledons and viridiplantae genes sets

	**Orthologs datasets**	
	Viridiplantae_odb10	Eudicotyledons_odb10
Complete BUSCOs	241 (56.1%)	1169 (55.2%)
Complete and single-copy BUSCOs	113 (26.3%)	521 (24.6%)
Complete and duplicated BUSCOs	128 (29.8%)	648 (30.6%)
Fragmented BUSCOs	141 (32.8%)	526 (24.8%)
Missing BUSCOs	48 (11.1%)	426 (20.0%)
Total BUSCO groups searched	430	2121

Table S2: Summary of sequencing data obtained from stems of *S. undatus*.

Sample ID	Location	Total PE Reads
MO1-1	Mozomboa, Veracruz	50,950,531
MO1-4	Mozomboa, Veracruz	36,294,903
MO2A	Mozomboa, Veracruz	38,977,914
MO2D	Mozomboa, Veracruz	40,606,094
HNC-2	Hunucmá, Yucatán	38,333,077
HNC-3	Hunucmá, Yucatán	37,643,845
UMN1	Umán, Yucatán	46,686,003
UMN2	Umán, Yucatán	51,133,696
